# A versatile modular vector set for optimizing protein expression among bacterial, yeast, insect and mammalian hosts

**DOI:** 10.1371/journal.pone.0227110

**Published:** 2019-12-30

**Authors:** Márk Somogyi, Tamás Szimler, Attila Baksa, Barbara M. Végh, Tamás Bakos, Katalin Paréj, Csaba Ádám, Áron Zsigmond, Márton Megyeri, Beáta Flachner, Ráchel Sajó, Éva Gráczer, Péter Závodszky, István Hajdú, László Beinrohr

**Affiliations:** Institute of Enzymology, Research Centre for Natural Sciences, Hungarian Academy of Sciences, Pázmány Péter sétány, Budapest, Hungary; Instituto Butantan, BRAZIL

## Abstract

We have developed a unified, versatile vector set for expression of recombinant proteins, fit for use in any bacterial, yeast, insect or mammalian cell host. The advantage of this system is its versatility at the vector level, achieved by the introduction of a novel expression cassette. This cassette contains a unified multi-cloning site, affinity tags, protease cleavable linkers, an optional secretion signal, and common restriction endonuclease sites at key positions. This way, genes of interest and all elements of the cassette can be switched freely among the vectors, using restriction digestion and ligation without the need of polymerase chain reaction (PCR). This vector set allows rapid protein expression screening of various hosts and affinity tags. The reason behind this approach was that it is difficult to predict which expression host and which affinity tag will lead to functional expression. The new system is based on four optimized and frequently used expression systems (*Escherichia coli* pET, the yeast *Pichia pastoris*, pVL and pIEx for *Spodoptera frugiperda* insect cells and pLEXm based mammalian systems), which were modified as described above. The resulting vector set was named pONE series. We have successfully applied the pONE vector set for expression of the following human proteins: the tumour suppressor RASSF1A and the protein kinases Aurora A and LIMK1. Finally, we used it to express the large multidomain protein, Rho-associated protein kinase 2 (ROCK2, 164 kDa) and demonstrated that the yeast *Pichia pastoris* reproducibly expresses the large ROCK2 kinase with identical activity to the insect cell produced counterpart. To our knowledge this is among the largest proteins ever expressed in yeast. This demonstrates that the cost-effective yeast system can match and replace the industry-standard insect cell expression system even for large and complex mammalian proteins. These experiments demonstrate the applicability of our pONE vector set.

## Introduction

Finding the optimal host for the expression of proteins for research purposes often involves expensive and time-consuming testing in multiple expression systems. Since different vectors were developed independently [1–3], they are usually not compatible, holding a different set of restriction sites. This means that in many cases the gene of interest needs to be re-cloned into each vector to be tried, even if the gene itself is unchanged. The expression screening process can be sped up by the use of vectors tailored/designed specifically for quick exchange of genes of interest. One such approach is the pAUL vector system developed for *Arabidopsis thaliana* [4]. There is also the pCri System with vectors for *E*. *coli*, *B*. *subtilis* and *P*. *pastoris* [5]. Other systems are aimed to handle different problems (e.g. gene-delivery [6], recombinant antibody expression [7] or metabolic engineering in yeast [8].

**Fig 1:**
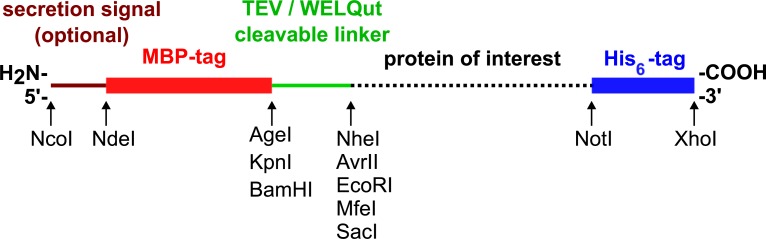
Features of the expression casette in the pONE vector set. The expression cassette begins with an N-terminal secretion signal, followed by a sequence of MBP affinity tag, a protease cleavable linker, the multiple cloning site, and finally a C-terminal His-tag. All of these elements are exchangeable among the vectors, provided the restriction sites are compatible. Many, but not all, restriction sites are compatible among the vectors. There is at least one appropriate restriction enzyme combination for each possible pair of vectors. ***Minimal requirements that make a vector “pONE” compatible***: NcoI and flanking NotI and XhoI sites. At least one of AgeI/KpnI/BamHI sites at the affinity tag/cleavage tag boundary. One of the NheI/AvrII pair. One of the EcoRI/MfeI pair. The pairs generate compatible overhangs, therefore they may be ligated together. (**A**) Schematic of the multiple cloning site. (**B**) Sequence details of the intracellular MBP-TEV variant of vectors. (**C**) Sequence details of the secreted MBP-WELQ variant of vectors.

In this paper we describe a new set of expression vectors, unified for a wide variety of host cells, such as *E*. *coli* (bacterium), *P*. *pastoris* (yeast), *S*. *frugiperda* (insect), and mammalian cells (human embryonic kidney [HEK] and chinese hamster ovary [CHO]). These vectors allow rapid swapping of genetic elements among themselves: they carry a unified multiple cloning site (Fig 1), enabling quick transfer of the target gene from one vector to another by simple excision and ligation. This arrangement also allows modular exchange of secretion signals, affinity tags and protease cleavable linkers among them. Here we describe the development process, involving the reduction or elimination of several restriction sites from the backbone of the vectors. In addition, we demonstrate the functionality of the modified vector set by expressing the maltose binding protein (MBP), often used for affinity labelling, as a reporter protein.

Finally, we show the usefulness of the vector set through its application to a practical problem: expression screening of a large human protein kinase, ROCK2, a large multidomain and multifunctional protein. Disturbances in ROCK2 function are associated with neurodegenerative diseases [9]. ROCK2 has been successfully expressed in the past in insect cell cultures [10]. Our expression screening reveals that ROCK2 kinase expressed in *P*. *pastoris* is functionally equivalent to that produced using insect cells. We also managed to purify the protein. To our knowledge, ROCK2 may well be the largest (a dimer of two 164 kDa chains) protein successfully expressed and purified from yeast so far. For comparison only the expression of the complex α_3_β_1_ isoform of Na, K ATPase (chain sizes of 110 and 35 kDa, respectively) may be mentioned [11]. The successful production of ROCK2 is an important achievement, since yeast expression systems [2] are cost-effective and scalable. Furthermore, we have demonstrated successful expression of other important human kinases, namely LIM domain kinase 1 (LIMK1) [12] and Aurora kinase A [13] as well as a tumour suppressor protein, Ras association domain-containing protein 1A (RASSF1A) (e.g. [14, 15] by the aid of the elaborated pONE vector set.

## Materials and methods

### Modification of expression plasmids, construction of pONE vectors

**Fig 2:**
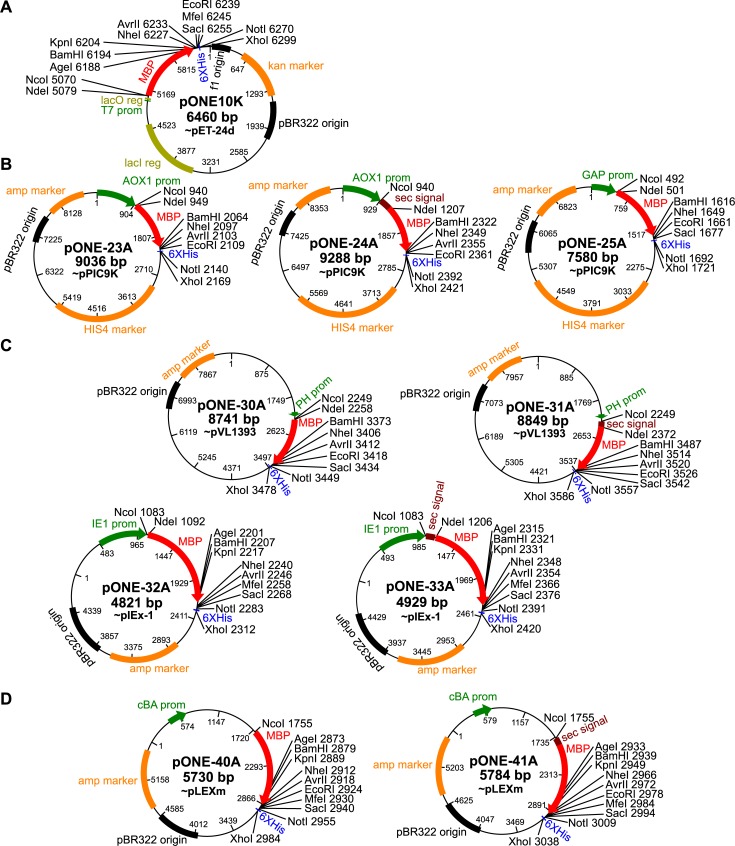
Maps of the pONE vectors. Promoters (green) and the MBP gene (red) in each vector are represented by arrows showing the direction of transcription and translation. Other important elements, depicted as rectangles are: 6X histidine tag (blue), resistance markers (orange) and replication origins (black). Secretion vectors also have genes encoding secretion signals (brown) in fusion with MBP. Important, unique restriction sites of the expression cassette are also marked. (**A**) The only *E*. *coli* vector of the set, pONE-10K carries the lacI and lacO elements of the lac operon (yellow). (**B**) *Pichia pastoris* vectors: pONE-23A (intracellular) and pONE-24A (secretion) have the AOX1 promoter, while pONE-25A (intracellular) utilizes the GAP promoter. (**C**) Insect cell vectors: pONE-30A (intracellular) and pONE-31A (secretion) are based on the baculovirus expression system, while pONE-32A (intracellular) and pONE-33A (secretion) can be used for transient transfection. (**D**) Mammalian vectors, fit for transient transfection: pONE-40A (intracellular) and pONE-41A (secretion). Vector maps were produced using BioEdit [19].

Modification and assembly of recombinant expression plasmids involved standard laboratory practices: polymerase chain reaction, PCR based mutagenesis, restriction digestion and ligation. Relevant protocols are available [16–18]. To unify the multiple cloning sites of the vectors, several restriction sites were removed from the vector backbones using mutagenesis or by a procedure involving restriction digestion of the vector, followed by overhang filling and finally, religation of the vector via the newly formed blunt ends. Sequences encoding for certain affinity tags, secretion signals and protease cleavage sites were also cloned into the vectors, yielding the “pONE” series of vectors. The signal sequence and tags are also separated by well-placed restriction sites for easy exchange between different signals and tags (Fig 1). Despite our efforts, not all restriction sites may work in all vectors, however, there is at least one set of restriction sites that will be unique. The usable (unique) restriction sites are marked in Fig 2 for each of the vectors. For these vectors (Table 1) we propose an easy-to-remember nomenclature, using the following logic: all names begin with pONE-, followed by a number with its first digit denoting the host (1: bacteria, 2: yeast, 3: insect and 4: mammalian)–the second digit is for distinguishing between the individual vectors. Vector names are terminated by a letter marking the antibiotic resistance in the vector (A: ampicillin, K: kanamycin). The resulting vector sequences are available in the supplemental data (S1 Sequences).

**Table 1:** The list of unified vectors of the pONE series.

Vector name	Host cell	Parental vector	Final vector composition	Type of vector	Resistance marker	Vector replication
pONE-10K	*E*. *coli*	pET-24d	pET-MBP-TEV-His6	intracellular	Kanamycin	replicative
pONE-23A	*P*. *pastoris*	pPIC9K	pPIC-MBP-TEV-His6	intracellular	Ampicillin/none (metabolic selection in yeast)	integrative
pONE-24A			pPIC-sec-MBP-WELQut-His6	secretion		
pONE-25A			pPIC-GAP-MBP-TEV-His6	intracellular		
pONE-30A	*S*. *frugiperda*	pVL1393	pVL-MBP-TEV-His6	intracellular	Ampicillin	replicative (virus)
pONE-31A			pVL-sec-MBP-WELQut-His6	secretion		
pONE-32A		pIEx-1	pIEx-MBP-TEV-His6	intracellular		transient
pONE-33A			pIEx-sec-MBP-WELQut-His6	secretion		
pONE-40A	*H*. *sapiens*	pLEXm	pLEX-MBP-TEV-His6	intracellular	Ampicillin	transient
pONE-41A			pLEX-sec-MBP-WELQut-His6	secretion		

### Testing the pONE vectors in protein expression experiments

Protein expression was carried out using standard procedures described in the relevant manufacturer/developer supplied manuals for the *Escherichia coli* pET system [20], transient expression system of insect cells [21], baculovirus-insect cell system [22] and the *Pichia* classic system [23]. The mammalian transient expression system was based on the pLEXm vector established by Cambridge researchers [1].

To examine protein expression levels among the different vectors, MBP (Maltose Binding Protein) was expressed in itself, and protein yields were compared for each vector using Western blot and densitometry. In addition, to test the vectors in a real-life project, the expression levels of the complex full-length human ROCK2 kinase were also compared in the vectors pONE-10K, -25A, -30A. The genes coding for the proteins were synthetized by Thermo Fisher Scientific GeneArt using the codon preference of insect cells. The sequences are provided in S1 Sequences. Other proteins were also expressed successfully using pONE vectors: pONE-10K proved efficient for expression and purification of Aurora kinase A and various truncated forms of Ras association domain-containing protein 1A (RASSF1A), as well as galectins 13 and 14. LIM domain kinase 1 (LIMK1) was expressed using pONE-30A.

#### Expression trials in *Escherichia coli*

For protein expression in bacteria, *E*. *coli* BL21 (DE3) cells were transformed with the appropriate plasmids and plated on antibiotic-containing Luria-Bertani broth (LB; plus 50 μg/mL kanamycin or 100 μg/mL ampicillin). A selected colony was inoculated in 20 mL LB medium and incubated overnight at 37°C, 220 rpm in the presence of an antibiotic. Next day, 1 mL or 10 mL of the starter culture was used to inoculate 100 mL or 1 L of LB broth respectively and cells were grown at 37°C, 220 rpm in the presence of an antibiotic. Protein expression was induced by adding 1 mM IPTG to the cell cultures, upon reaching an OD 600 density of ~0.8–0.9. Before induction, cultures were equilibrated to induction temperature 30°C for 0.5 hours. Protein expression was continued for 4 hours at 30°C, 220 rpm.

#### Expression trials in *Pichia pastoris*

For expression in *P*. *pastoris*, recombinant protein containing vectors were linearized using PmeI or AvrII restriction enzyme–depending on the vector–and transformed via electroporation. Cleaved vector DNA targets the vector for integration into the yeast genome at the cleavage region. Without cleavage transformation efficiency is low and may result in non-expressing clones. PmeI site is present in the vectors with AOX1 promoter, while AvrII is present in the GAP promoter. It should be noted that AvrII is not unique, however the other AvrII site is eliminated in most cloning strategies. If not, the unique BglII site may be used, albeit this results in lower transformation efficiency. Cells were plated on Minimal Dextrose (MD) plates (for vectors with HIS4 metabolic marker) and incubated for 3 days at 30°C. Freshly grown colonies from the plates were picked and grown in 20 mL or 3×100 mL YPD medium at 30°C overnight at 280 rpm. The pre-inoculum was used to inoculate 200 mL or 3×1 L Buffered Media with Glycerol (BMGY) medium and were grown for 8 hours at 30°C at 280 rpm in Erlenmeyer flasks. The flasks were not filled with more media than 25% of nominal volume.

#### Expression trials in *Spodoptera frugiperda*

For the pVL based vectors pONE-30A and -31A, Sf9 insect cells were co-transfected with expression plasmid and linearized baculovirus DNA (Oxford Expression Technologies) in a 6-well plate. Baculovirus was amplified in two further rounds (p1 and p2) as a monolayer culture to produce a virus stock ready for infection of the expression cells. Sf9 insect cells at 2×10^6^ cell/mL density were infected with recombinant baculovirus to produce the protein of interest in 2 mL or 500 mL volume in Insect-Xpress medium (Lonza). The infected cells were incubated for 3 days at 27°C with constant shaking at 110 rpm.

For transient transfection of Sf9 cells, pIEx based vectors pONE-32A and -33A were mixed with Insect GeneJuice transfection reagent (Sigma-Aldrich) and added to cell cultures of 2×10^6^ cell/mL density, as described in the Insect GeneJuice datasheet [24]. Transfected cells were incubated for 72 hours at 27°C, before further processing.

#### Expression trials in the human cell line HEK293

HEK293 mammalian cells were transfected with the mixture of branched polyethylene-imine (No. 408727, Sigma-Aldrich**)** and pONE-40A (or pONE-41A) constructs (ratio 3:1) at 5×10^5^ cell/mL density in 2 mL Dulbecco’s Modified Eagle’s Medium containing 10% FBS (Fetal Bovine Serum) and penicillin-streptomycin antibiotic. Transfected cells were incubated for 72 hours at 37°C in the presence of 5% carbon-dioxide. After incubation, cells were separated from the medium for further analysis.

#### Purification of the recombinant proteins

After expression, cells were separated from the expression medium by centrifugation (10 min, 16000 g for *E*. *coli*, 4000 g for other hosts). Proteins of interest were localised in different fractions depending on the expression method. Proteins without a secretion signal accumulated inside the cytoplasm and had to be extracted from the cells: The cells were resuspended in a buffer (25 mM HEPES, 500 mM NaCl, 10 mM β-mercaptoethanol, 1 mM PMSF, pH 7.4). Then, cells were lysed via ultrasonic homogenization. Cellular debris was pelleted using centrifugation (45 min, 44000 g), and the protein of interest purified from the cellular supernatant.

Proteins expressed in fusion with a secretion signal appeared in the expression medium and could be purified directly from it.

Before purification, cellular extract or the expression medium containing the protein of interest was filtered through a syringe filter with a pore size of 0.2 μm. All proteins were purified using amylose affinity chromatography. Proteins of interest were eluted by 10 mM maltose, and peak fractions were collected. Aurora A protein was subjected to Ni affinity chromatography using 300 mM imidazole as eluent. Both Aurora A and RASSF1A were further purified by size exclusion chromatography on a column loaded with preparative grade Superose 12 resin.

#### Determination of protein expression yields

After purification, protein concentrations were determined by UV spectrophotometry using molar extinction coefficients calculated on the basis of a previously published method [25]. Total protein yields for each batch were calculated from the concentration data and the molar weights of the recombinant proteins. Yields were normalized for wet cellular mass measured during each expression.

#### SDS-PAGE and Western blot analysis

Protein samples were analysed by SDS-PAGE on gels containing 7.5% or 10% acrylamide. The gels were either stained with Coomassie brilliant blue, or further used for Western blotting.

For Western blot analysis, proteins separated by SDS-PAGE were transferred to a nitrocellulose membrane (Bio-Rad) and were blocked by overnight incubation with 10 mL blocking buffer (20 mM Tris, 200 mM NaCl, 0.02% NaN_3_, pH 7.2) containing 1.5% casein. Proteins were detected via their MBP fusion tag, by immunoblot analysis using a primary antibody (Rb anti-Maltose Binding Protein polyclonal antibody, Abcam) at a dilution of 1:3500 and a secondary antibody (Ms anti-rabbit IgG (γ-chain specific)-peroxidase-conjugated monoclonal antibody, Sigma) at a dilution of 1:10000 in blocking solution. The protein-antibody complexes were detected utilizing a chemiluminescence system (Novex ECL HRP Chemiluminescent Substrate Reagent Kit, Invitrogen). The membranes were exposed for 3 seconds, and images were evaluated using ImageJ software [26] to determine band density. Protein amounts were calculated from density data with the aid of a calibration series of known amounts of purified MBP.

In the case of ROCK2, alkaline phosphatase-conjugated secondary antibody (Gt anti-rabbit IgG, Sigma) was also used at dilution of 1:3000 in blocking solution. After incubation, a mixture of nitro-blue tetrazolium (NBT) and 5-bromo-4-chloro-3'-indolyphosphate (BCIP) was used for antibody complex detection.

### Protein functional analysis

Kinase activities were determined using a Z’-LYTE Peptide Kinase Assay [27], with substrates Ser/Thr peptide 1 for Aurora A and Ser/Thr peptide 13 for other kinases. Reaction buffer contained 50 mM HEPES (pH 7.5), 10 mM MgCl_2_, 1 mM EGTA, 0.01% Brij-35, 1% DMSO, 20 μM ATP and 2 μM peptide substrate. The kinase reactions were incubated at 30°C for 1 hour, followed by development by the included protease at 25°C for 1 hour. Fluorescence emissions were detected at 445 nm and 520 nm using an EnSpire Multimode Plate Reader. Based on the emission values, and by using phosphorylation control solutions, phosphorylation amounts for each reaction were calculated using the formula provided in the Z’-Lyte manual [28]. Phosphorylation percentage values were directly converted to concentrations. Reactions with no more than 10% substrate consumption could be assumed to be in the linear kinetics stage. Initial velocities were calculated from these data. We have obtained normalised activity values by dividing these initial velocities by the concentration of the enzyme (expressed in monomers).

## Results and discussion

### Vector design

Our modular, unified vector system, named pONE permits a one-step exchange of the gene of interest among the vectors for recombinant protein expression in bacteria (*E*. *coli*), yeast (*P*. *pastoris*), insect cell (*S*. *frugiperda*) and mammalian cells (HEK293 or CHO). This compatibility is ensured by an expression cassette with its multiple cloning site unified between all pONE vectors (Fig 1). The multiple cloning site is not totally identical in all vectors, but for any pair of vectors selected, there exists at least one compatible combination of restriction sites, which might be used for direct cloning (Fig 2). The expression cassette (Fig 1) also has genes for MBP (N-terminal) and His_6_ (C-terminal) fusion affinity tags, and an N-terminal secretion signal may be included, too (before the MBP-tag).

MBP was chosen because it can be used directly in affinity purification schemes using relatively inexpensive, common chromatographic media and it is widely regarded as a protein folding chaperone that increases expression level of proteins fused to it (demonstrated in *E*. *coli* and even in *P*. *pastoris* [29]).

The linker between the MBP and the target gene encodes the recognition site for either a Tobacco Etch Virus protease (TEV [30, 31]) or a WELQut [32] protease, allowing the removal of the large MBP tag from the protein product. The length and composition of the linker can be fine-tuned by the careful selection of restriction sites for cloning. For example, the protease cleavage site, a short hydrophobic streak or a Pro-Arg pair (providing some rigidity) can be excluded or included depending on this choice. The expression cassette is modular in nature, meaning that all auxiliary genes (affinity tags, secretion signal, and protease cleavage site) are flanked by restriction sites, so they can be replaced or removed easily.

The pONE vector set contains bacterial, yeast, insect cell (baculoviral and transient) and mammalian vectors, which are based on the commercially available pET-24d [20], pPIC9K [23], pVL1393 [22], pIEx-1 [21] and pLEXm [1] vectors, respectively (Table 1 and Fig 2). These vectors were selected because they are regarded as some of the best expression vectors available. Most of these vectors were created in two variants: one including a secretion signal specific for its host, and one without a secretion signal. The secretion variants have WELQut, while the intracellular ones have TEV protease cleavage sites.

The protease choice was dictated by the buffers: TEV protease is a cysteine protease which needs reducing environment for catalytic activity. The WELQut protease functions well in oxidising environments too, because it is a serine protease, and therefore not redox-sensitive. Intracellular cytosolic proteins often have free cysteines and require reducing conditions *in vitro* to keep them active and prevent their aggregation. Secreted proteins are usually transferred into the extracellular space, and often have disulphide bonds required for their structural stability. The reducing TEV protease buffers present a risk for these disulphide bridges. The non-reducing WELQut protease buffers are better suited for redox-sensitive proteins.

The bacterial vector uses the T7 promoter, enabling powerful induction of protein expression upon addition of IPTG. The *Pichia* expression systems traditionally utilize the AOX1 promoter, which can be induced by the addition of methanol when no other carbon source is present. We have also created a *Pichia* vector (pONE-25A), which has a constitutively active GAP promoter. GAP promoter eliminates the need for using methanol and is less stressful for the cells, which is an advantage in some situations, especially in large-scale applications. The pIEX (pONE-32A and -33A) and pLEXm (-40A and -41A) based vectors can be used for transient transfection of insect and mammalian hosts, respectively, therefore they use constitutive promoters too. For insect cells, baculovirus-based vectors (pONE-30A and -31A) were also constructed.

### Trial expressions with blank vectors

**Fig 3:**
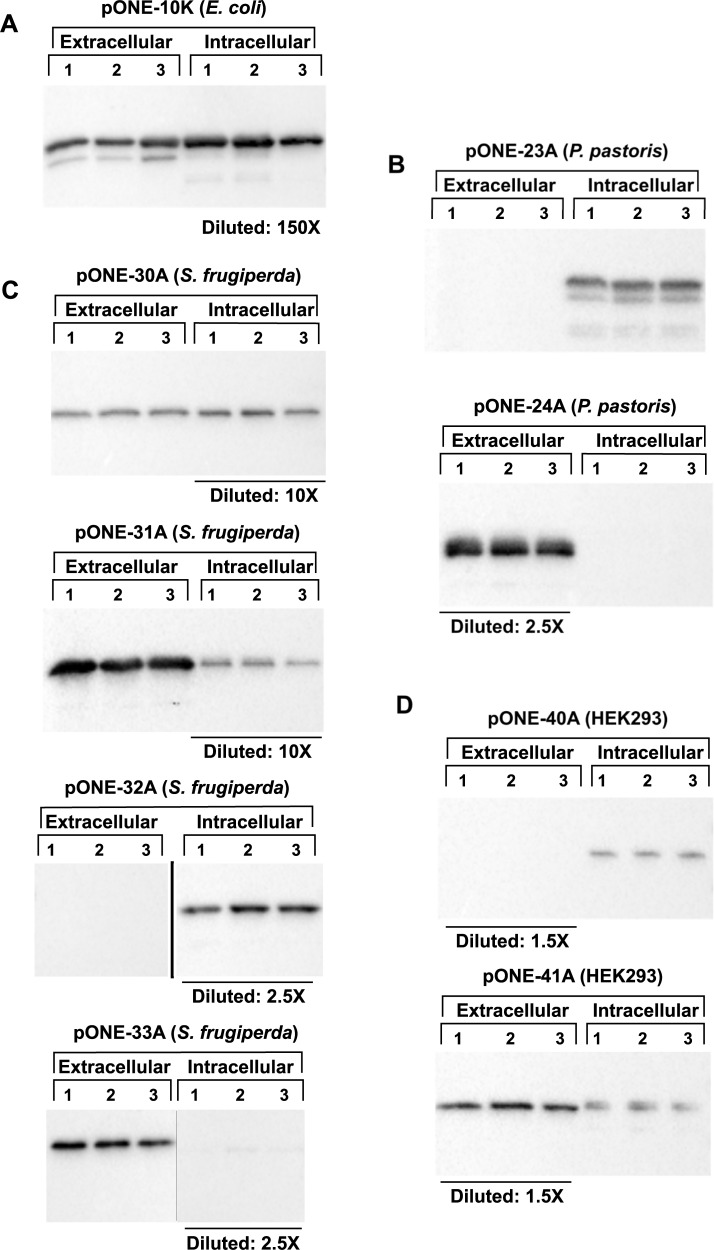
Expression patterns of MBP using pONE vectors demonstrate that proteins of interest are expressed and directed to the relevant compartment. Recombinant MBP (~45 kDa) was expressed in various hosts and pONE vectors, as described in Materials and Methods. Three parallel experiments were performed for each vector (marked by numbers 1–3). Samples were taken from the expression media (‘Extracellular’) and cell lysates (‘Intracellular’), then analysed via SDS PAGE followed by Western blotting. Relative dilutions (each valid within a given blot) are marked below the corresponding lanes. An anti-MBP antibody was used for detection of the recombinant protein. Blots were developed in a bioluminescent reaction (cf. Materials and Methods). Band intensities were determined by densitometry, and converted to total protein mass using internal calibration standards on each blot, with known amounts of MBP. Distribution of MBP between the extra- and intracellular compartments is presented in Table 2. (**A**) pONE-10K is a vector for intracellular protein expression in *E*. *coli*. Considering the sample dilutions, protein leakage is negligible. An MBP form of smaller molecular mass is also present, especially in the extracellular samples. This is likely the form natively expressed by *E*. *coli*, which has a molar weight of ~43 kDa (our recombinant variant is ~45 kDa). (**B**) MBP expression in *P*. *pastoris* using the pONE-23A (intracellular) and pONE-24A (secretion) vectors. Some inhomogeneities are detectable in the intracellular samples. These are most probably degradation products, resulting from protease activity in the cytoplasm. (**C**) pONE-30A (intracellular) and pONE-31A (secretion) are baculovirus based vectors for *S*. *frugiperda* cells, while pONE-32A (intracellular) and pONE-33A (secretion) are used for transient transfection. No degradation is observed. MBP targeting works well, with the probable exception of pONE-31A, where approximately half of the product is held back in the cell. (**D**) MBP expression in HEK293 cells, using the pLEXm based pONE-40A (intracellular) and pONE-41A (secretion) vectors. Intracellular retention of some MBP with the pONE-41A vector can be observed, this is probably misfolded material.

To test the functionality of pONE vectors, we have performed various trial expressions. In the first series of experiments, “empty” vectors were tested to check whether the modifications did affect their performance, and also to see if the protein is targeted properly in the presence or absence of a secretion signal. Since all components of the expression cassette are in frame (Fig 1), the “empty” vectors yield fusion protein products, with MBP as their major part. These experiments were performed with all vectors, excluding pONE-25A, which was tested by expressing ROCK2 kinase (cf. next chapter). After expression, total protein content of the cells and expression media were analysed–the recombinant product was detected via Western blot using an MBP-specific antibody. The resulting blot images, with three parallel experiments for each pONE construct are presented in Fig 3. From these data the relative distribution of MBP between the medium and cytoplasm was calculated for each vector and are summarized in Table 2.

**Table 2:** Expression yields and localization of recombinant MBP in various hosts. Western blots (Fig 3) were subjected to densitometric analysis using ImageJ software [26]. Results were quantified using calibration standards on each Western blot. From these data relative distributions of MBP between the extracellular and intracellular compartments were calculated. “nd” means “not detected”. Expression patterns of MBP using pONE vectors demonstrate that proteins of interest are expressed and directed to the relevant compartment.

Host	Vector	Distribution of MBP between compartments	
		Extracellular	Intracellular
Bacteria	pONE-10K	<1%	>99%
Yeast	pONE-23A	nd	100%
	pONE-24A	100%	nd
Insect	pONE-30A	8 ±1%	92 ±1%
	pONE-31A	49 ±10%	51 ±10%
	pONE-32A	nd	100%
	pONE-33A	96 ± 1%	4 ±1%
Mammal	pONE-40A	nd	100%
	pONE-41A	82 ±3%	18 ±3%

These experiments prove that all pONE vectors are functional–significant quantities of MBP were produced by each expression system (Fig 3). In general, it is in line with those observations, that found MBP is a useful and expression enhancing tag even in higher eukaryotes [33–36]. The variations between parallel experiments are relatively small, indicating reproducibility of expression. Protein targeting works very well–although in the case of the pONE-31A (and probably -41A) secretion vectors pronounced intracellular protein retention could be detected. This could be explained by aggregation of the overexpressed protein, which reduces secretion efficiency. For pONE-31A another possible cause might be the blockage of the endoplasmic reticulum by baculoviral proteins.

### Expression of a complex protein kinase, ROCK2

In the next phase of the project, we applied the pONE vectors to a real-life problem. To study the activation mechanism of ROCK2, a large size (molecular mass of 164 kDa) multidomain, multifunctional kinase, associated with neurodegenerative diseases [9, 37], by *in vitro* experiments we aimed to develop a reproducible protocol for its expression and purification. Therefore, to determine which provides the most functional protein, we have performed a systematic expression screen of ROCK2 in expression hosts *E*. *coli*, *P*. *pastoris* and the baculovirus-insect cell system. As ROCK2 has successfully been produced in insect cells in the past [10], we expected this host to be the most suitable one.

**Fig 4:**
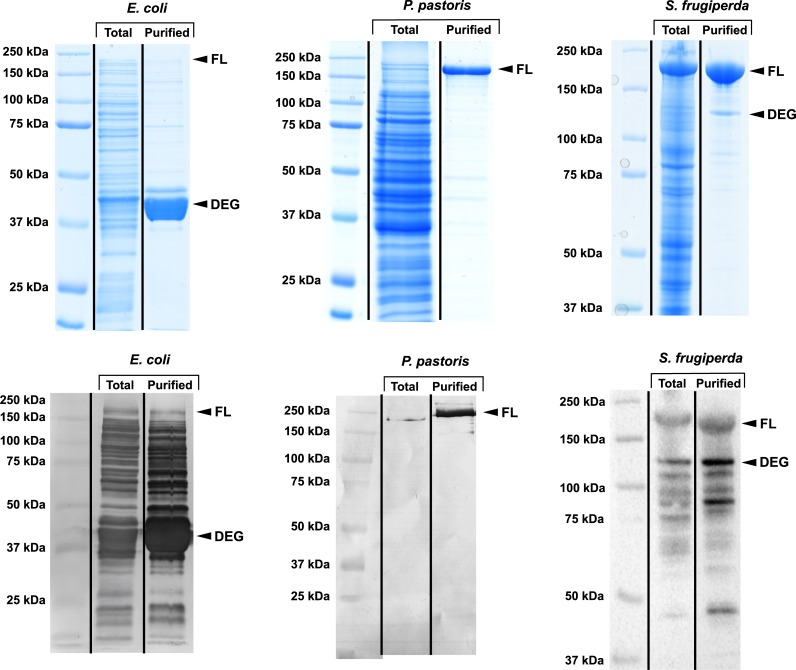
ROCK2 expression and purification in various hosts. To test the pONE vector set we have used it for the expression screening of a large, multi-domain human protein, the ROCK2 kinase. ROCK2 was expressed in the bacterium *E*. *coli*, the yeast *P*. *pastoris* and the insect cell *S*. *frugiperda* using the intracellular vectors pONE-10K, pONE25-A and pONE30-A, respectively. Cells were lysed and ROCK2 purified from the lysate by amylose affinity chromatography. Samples were taken during the process, and analysed by SDS PAGE and Western blotting using anti-MBP antibody (upper and lower images, respectively). As for the evaluation of the results, it is notable that impurities are generally more visible on Western blots due to its greater sensitivity and non-linearity compared to Coomassie staining. Each image presents the total protein content after expression (‘Total’) and the purified protein after the affinity chromatography (‘Purified’). Bands corresponding to full-length ROCK2 (‘FL’, M_w_ ~206 kDa with the fusion MBP tag) and the main degradation product (‘DEG’) are marked. *E*. *coli* is not suitable for ROCK2 expression, as the amount of full-length protein expressed is negligible compared to the degradation products, likely due to folding problems. In this host, the ROCK2 segment (M_w_ ~161 kDa) of the fusion protein degrades almost completely, while MBP (M_w_ ~45 kDa) remains intact, probably protected by its correct fold. In eukaryotic hosts, however, the bulk of ROCK2 is expressed as its full-length form. ROCK2 expressed in *P*. *pastoris* proved to be at least as pure as that expressed in *S*. *frugiperda*, the most commonly used host for ROCK2 expression. In fact, the presence of degradation products appears to be lower than in insect cells. However, both in *P*. *pastoris* and *S*. *frugiperda* a small amount of impurities appear along with the purified protein. Based on the Western blots, these are host proteins in the case of *P*. *pastoris*, and degradation products of ROCK2 in the case of *S*. *frugiperda*. In insect cells the degradation product is probably a fragmented MBP-ROCK2 fusion protein, where the C-terminal domain(s) of ROCK2 are cleaved off. It is demonstrated that yeast can effectively substitute insect cell culture for the expression of large, complex, multi-domain proteins.

A variant of the ROCK2 gene, optimized for expression in insect cells, was synthesized for the expression screening. This gene was ligated into the intracellular vectors pONE-10K (*E*. *coli*), pONE-25A (*P*. *pastoris*) and pONE-30A (*S*. *frugiperda*). After expression, recombinant ROCK2 was purified from the cell lysates using amylose affinity chromatography. Samples were taken after both protein expression and purification for analysis by SDS-PAGE and Western blotting (Fig 4). Protein yields, normalized to wet cellular mass are presented in Table 3 –all data represent three parallel experiments. As a functional test of the recombinant proteins their enzymatic activities were also measured as described in Methods.

**Table 3:** Yields and activities for proteins expressed using pONE vectors. Purification yields and functionality of full-length human ROCK2, Aurora A and LIMK1 kinases as well as a RASSF1A fragment expressed by different hosts (as illustrated by Figs 4 and 5).

Protein	Host	Vector	Purified protein / 1g wet cell mass	Enzyme activity
ROCK2	*E*. *coli* (bacterium)	pONE-10K	1.2 ± 0.2 μg	-
	*P*. *pastoris* (yeast)	pONE-25A	21 ± 2 μg	+
	*S*. *frugiperda* (insect cells)	pONE-30A	300 ± 30 μg	+
RASSF1A fragment	*E*. *coli*	pONE-10K	235 ± 78 μg	*
Aurora A kinase domain	*E*. *coli*	pONE-10K	137 ± 45 μg	+
LIMK1	*S*. *frugiperda*	pONE-30A	750 ± 230 μg	+

+/- signs mark if the enzyme was found active.

* marks that RASSF1A inherently lacks enzyme activity.

Expression in insect cells (*S*. *frugiperda*) was robust (Fig 4), with negligible protein degradation (relative amounts are shown better by Coomassie staining than by Western blotting). In contrast, we have found that *E*. *coli* is ill-suited for ROCK2 expression, even when using the codon supplemented Rosetta2 strain. Most of the product is degraded or expressed truncated–the amount of full-length ROCK2 is minuscule. For expression in yeast cells (*P*. *pastoris*) we have utilized a protease-deficient strain SMD1168. Here ROCK2 was expressed under the GAP promoter. The product was at least as homogenous as that expressed by the baculovirus-insect cell system.

For functional comparison of the ROCK2 products expressed in different hosts we have performed enzyme activity measurements using a Z’-Lyte assay kit (cf. Materials and methods). We cannot be sure if the substrate concentrations used in these experiments (especially the peptide at 2 μM) are at saturation. Because the conditions were identical in all reactions, the resulting activities are suitable for comparison between ROCK2 forms expressed in various hosts. Activities obtained were 0.03 ±0.01, 1.44 ±0.73, 1.05 ±0.38 [*mole product*] / ([*mole enzyme*] * min) for *E*. *coli*, *S*. *frugiperda* and *P*. *pastoris*, in the same order. These data also support that *E*. *coli* is unfit for ROCK2 expression: this product proved practically inactive. Insect cell- and yeast-produced ROCK2 functioned much better, with comparable activities.

Our experiments demonstrate that ROCK2 purified from yeast cells is functionally equivalent to the protein expressed in insect cells. Expression in the insect cells (*S*. *frugiperda*) still yields more ROCK2 per unit of cellular mass compared to yeast (*P*. *pastoris*). However, considering that protein expression in yeast is cheaper and much more scalable than in insect cells, *P*. *pastoris* could be, in many cases, a better and more economical choice for ROCK2 production, especially if mutagenesis studies are to be performed.

### Further examples for application of the pONE vector set

**Fig 5:**
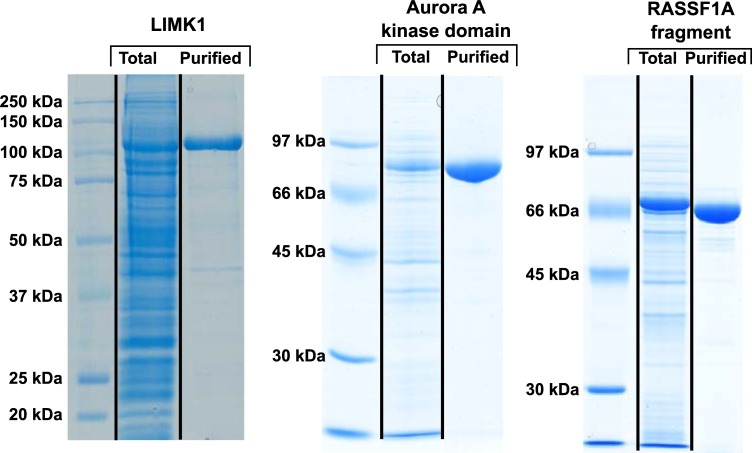
Purity of various protein constructs expressed using pONE vectors. The pONE vector set was also applied to express the following proteins. Full-length LIMK1 (M_w_ ~117 kDa) was expressed in the insect cells *S*. *frugiperda*, using the vector pONE-30A. The kinase domain of Aurora kinase A (M_w_ ~79 kDa) as well as a RASSF1A fragment (residues 121–340, M_w_ ~70 kDa) were both expressed in the bacterium *E*. *coli* using pONE-10K. All molecular masses correspond to the recombinant proteins in fusion with an MBP tag. Samples were taken before harvesting the cells (‘Total’) and from the final, purified protein product (‘Purified’). The samples were analysed by SDS-PAGE and subsequent staining by Coomassie brilliant blue. All proteins were expressed in substantial quantities (Table 3), and could be purified. Using pONE vectors, pure recombinant proteins (LIMK1, Aurora A kinase domain and a RASSF1A fragment) were obtained with high yield.

We have also successfully applied the pONE vector set to the expression of a number of other proteins. Namely, the tumour-suppressor RASSF1A, its oncogenic protein kinase, Aurora A [38]and another protein kinase, LIMK1. For these proteins, we do not show the expression trials, only the results of their expression and purification using the finally selected vectors (Fig 5, Table 3). Other examples for successful application of pONE vectors are described elsewhere: the expression of immunomodulators galectin-13 and -14 [39] and variants of UDP-glycosyltransferase [40]. The two galectins, the glycosyltransferase, the kinase domain of Aurora A and a deletion mutant of RASSF1A (with residues 121–340) could be obtained in a pure and functional form by expression in *E*. *coli* using the vector pONE-10K. LIMK1 was expressed in insect cells using the baculovirus based vector, pONE-30A. This enzyme could also be purified in an active, functional form.

### Comparison between the pONE vector set and existing expression systems

Expression vectors of different origins are rarely developed with compatibility in mind. This complicates the expression screening process often necessary when working with novel proteins. There are multiple vector systems designed to facilitate expression screening of various hosts, promoters or fusion tags. Some examples are the pBV boost [41], pDEP [42], pAUL [4] or pCri [5] systems. However, most systems are suitable only for a limited set of hosts, do not include variable auxiliary elements (expression tags, signal peptides), utilize complicated cloning techniques, or were developed for a specific task making them ill-suited for general research purposes.

The pONE system includes vectors for the four most widely used expression hosts: the bacterial, yeast, insect and mammalian systems. These vectors share a unified expression cassette, which allows rapid transfer of genes of interest between them. Cloning is achieved by the common restriction digestion/ligation method, which does not require expensive reagents or special knowledge, and is readily available at most research laboratories. The expression cassette also includes genes for MBP- and His_6_- fusion tags and in some vectors a secretion signal too. These are flanked by restriction sites and so can easily be removed or replaced by other sequences. The linker between MBP and the protein of interest includes a protease cleavage site, so MBP can be removed from the product easily. Also, the length and composition of the linker can be fine-tuned by selecting the appropriate restriction site for cloning. To sum up, the pONE vector set is a useful tool for general expression screening in a wide variety of host organisms, affinity tags and protein targeting, most suitable for small-scale research use.

## Conclusion

We have developed a new set of expression vectors, named the pONE series, suitable for protein expression in various, common host organisms. These vectors carry a unified, modular expression cassette, which allows rapid expression screening of novel proteins, and easy manipulation of fusion tags and signal peptides. Trial expressions of MBP protein carried by the “empty” vectors proves the functionality of all members of the vector set, including the proper targeting of recombinant proteins by an optional secretion signal. We have also demonstrated the usefulness of pONE vectors by applying them to the expression and purification of various complex proteins, namely ROCK2, LIMK1 and Aurora A kinases, the tumour suppressor RASSF1A and immunomodulators galectin-13 and -14. A significant finding of practical importance arose from the expression screening of ROCK2. Expression of this large, multi-domain protein in *P*. *pastoris* yielded a product with equal activity to that expressed in insect cell culture, which is commonly used for production of ROCK2. Since yeast-based expression systems are far more scalable than insect cell cultures, we expect that these results provide a promising alternative instead of the error-prone and costly insect cell expression.

## Supporting information

S1 Raw Images Raw originals of gel and Western blot images presented in the paper. Molar weights corresponding to bands of the molar weight marker (M) are listed near each image.
(PDF)

S1 Fig Functionality of the TEV protease cleavage site using MBP-ROCK2 fusion protein as example. MBP was successfully cleaved from an MBP-ROCK2 fusion product *in vitro* using TEV protease. ROCK2 at 5 μM concentration was incubated with 0.35 μM TEV protease [31] for 120 minutes at room temperature. The reaction buffer (pH 7.4) contained 25 mM HEPES, 150 mM NaCl, 1 mM DTT and 0.5 mM EDTA. Samples were taken (i) of the intact MBP-ROCK2 fusion protein (‘Fusion’), (ii) the reaction mixture after the incubation (‘Cleaved’) and (iii) the TEV protease in itself. The band corresponding to MBP-ROCK2 (M_w_ ~206 kDa) disappeared after the incubation, while two other bands appeared approximately at the weights of free ROCK2 (M_w_ ~161 kDa) and MBP (M_w_ ~45 kDa). This sample also contained TEV protease (M_w_ ~26 kDa).
(PDF)

S1 Sequences The vector and protein sequences described in this study in FASTA format. (ZIP)
