# Supplementary figures and images for: A versatile modular vector set for optimizing protein expression among bacterial, yeast, insect and mammalian hosts
Source: PLoS One. 2019 Dec 30;14(12):e0227110. doi: 10.1371/journal.pone.0227110 (PMC6936851; doi:10.1371/journal.pone.0227110)

Figure 3.

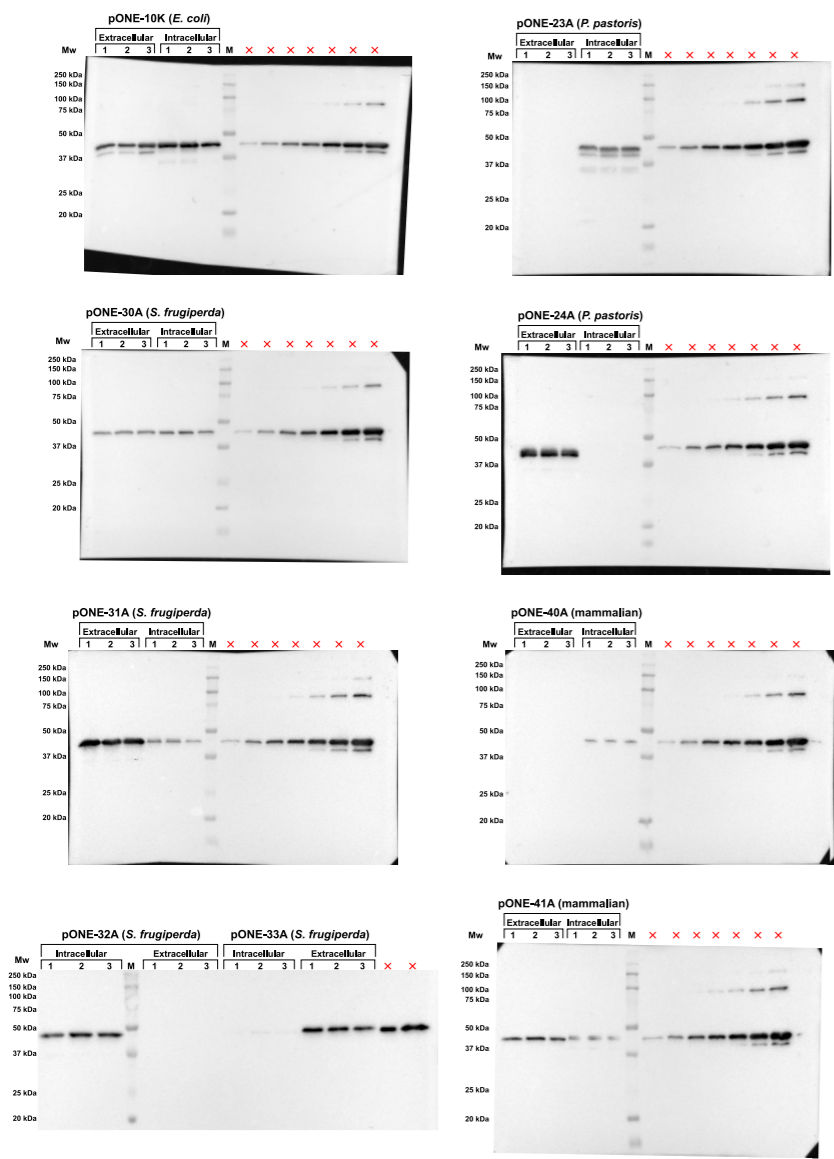

Figure 4.

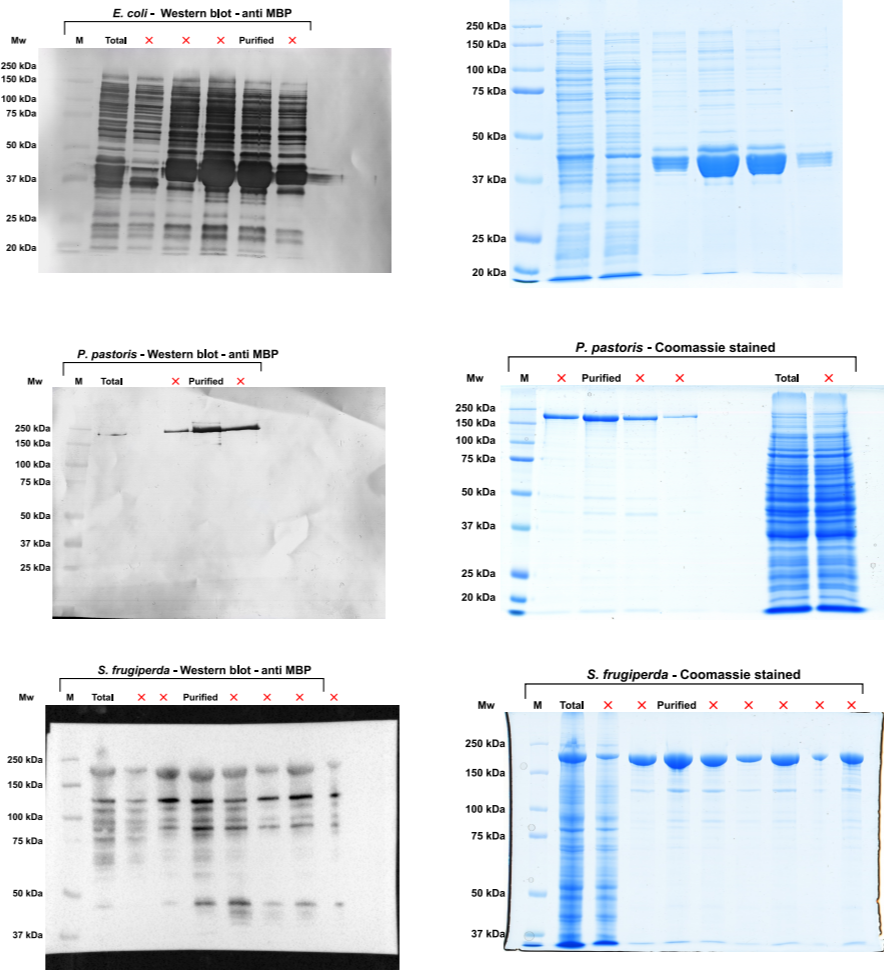

Figure 5.

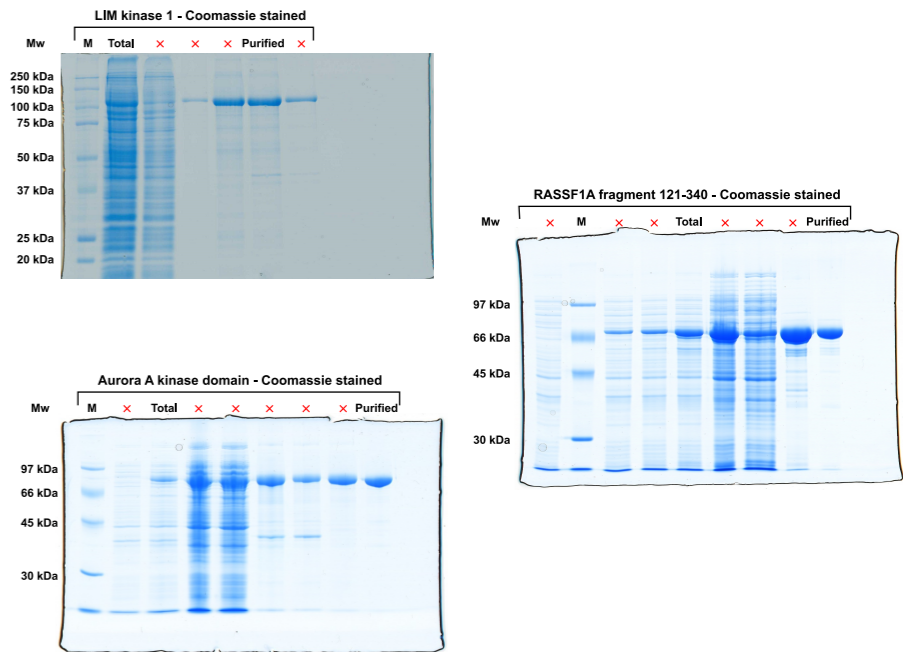

Supplementary Figure 2.

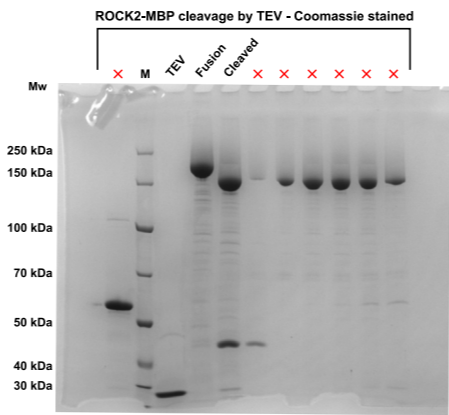

Supplement: S1 Raw Images — Molar weights corresponding to bands of the molar weight marker (M) are listed near each image. (PDF) [file pone.0227110.s001.pdf]

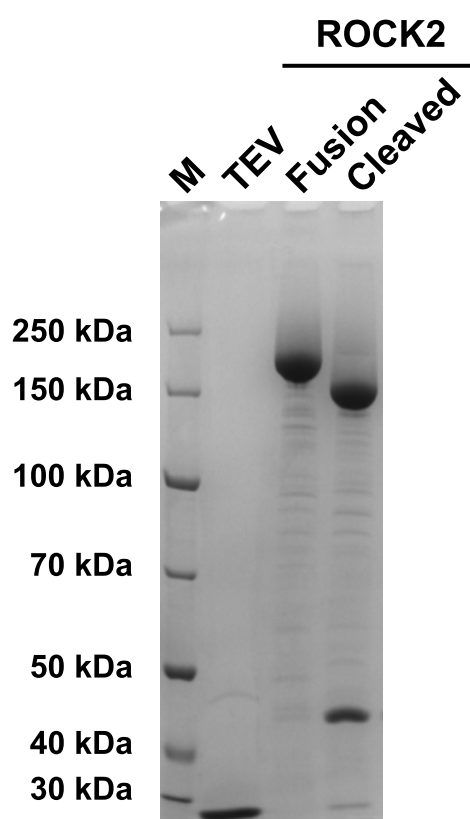

Supplement: S1 Fig — MBP was successfully cleaved from an MBP-ROCK2 fusion product in vitro using TEV protease. ROCK2 at 5 μM concentration was incubated with 0.35 μM TEV protease [31] for 120 minutes at room temperature. The reaction buffer (pH 7.4) contained 25 mM HEPES, 150 mM NaCl, 1 mM DTT and 0.5 mM EDTA. Samples were taken (i) of the intact MBP-ROCK2 fusion protein (‘Fusion’), (ii) the reaction mixture after the incubation (‘Cleaved’) and (iii) the TEV protease in itself. The band corresponding to MBP-ROCK2 (Mw ~206 kDa) disappeared after the incubation, while two other bands appeared approximately at the weights of free ROCK2 (Mw ~161 kDa) and MBP (Mw ~45 kDa). This sample also contained TEV protease (Mw ~26 kDa). (PDF) [file pone.0227110.s002.pdf]
